# Drug-Based Gold Nanoparticles Overgrowth for Enhanced SPR Biosensing of Doxycycline

**DOI:** 10.3390/bios10110184

**Published:** 2020-11-19

**Authors:** Syed Akif Raza Kazmi, Muhammad Zahid Qureshi, Jean-Francois Masson

**Affiliations:** 1 Department of Chemistry, Government College University Lahore, Lahore 54000, Pakistan; dr.zahidqureshi@gcu.edu.pk; 2 Département de Chimie, Québec Centre for Advanced Materials and Regroupement Québécois sur les Matériaux de Pointe, C.P 6128 Succursale Centre-Ville Université de Montréal, Montreal, QC H3C 3J7, Canada

**Keywords:** gold nanoparticles, doxycycline, tetracycline, SPR biosensor, signal amplification, clinical diagnosis

## Abstract

In clinical chemistry, frequent monitoring of drug levels in patients has gained considerable importance because of the benefits of drug monitoring on human health, such as the avoidance of high risk of over dosage or increased therapeutic efficacy. In this work, we demonstrate that the drug doxycycline can act as an Au nanoparticle (doxy-AuNP) growth and capping agent to enhance the response of a surface plasmon resonance (SPR) biosensor for this drug. SPR analysis revealed the high sensitivity of doxy-AuNPs towards the detection of free doxycycline. More specifically, doxy-AuNPs bound with protease-activated receptor-1 (PAR-1) immobilized on the SPR sensing surface yield the response in SPR, which was enhanced following the addition of free doxy (analyte) to the solution of doxy-AuNPs. This biosensor allowed for doxycycline detection at concentrations as low as 7 pM. The study also examined the role of colloidal stability and growth of doxy-AuNPs in relation to the response-enhancement strategy based on doxy-AuNPs. Thus, the doxy-AuNPs-based SPR biosensor is an excellent platform for the detection of doxycycline and demonstrates a new biosensing scheme where the analyte can provide enhancement.

## 1. Introduction

It is important in the analytical community to develop sensors for frequent monitoring of drugs. In particular, clinical diagnosis and therapeutic procedures demand sensors to assess drug levels in patients. There is a need to obtain accurate test results in a short time for a large number of samples to decide on the course of medical treatments [1]. As such, different laboratory-based techniques are often employed to measure the drug concentrations in biofluid of patients, which serves to adjust medication knowing the active concentration of drugs in blood. As such, regular monitoring of drug levels in patients can prevent its toxicity and damage to organs [2,3].

Doxycycline is a wide-spectrum drug belonging to the tetracycline family of antibiotics, which has been a drug of choice for treatment of several types of bacterial infections [4]. Protease-activated receptor-1 (PAR-1) is the member of PARs (protease-activated receptors) family that was identified in 1991 by two independent laboratories during the investigation of thrombin signaling pathway in both hamster and human cells. PAR-1 is expressed in all kinds of blood cells as well as in immune cells epithelium, astrocytes, and neurons [5]. PAR-1 is the receptor protein with which doxycycline binds to inhibit the tumor progression [6].

According to FDA, safe dose of doxycycline is 200 mg on first day of treatment followed by 100 mg per day. In case of prolonged high dose, patient may suffer from gastrointestinal and renal diseases. It is a relatively low-toxicity drug and has been recommended for human use for a long time. However, the long-term use of doxycycline may lead to some side effects. Elzeinová et al. reported the adverse effects of doxycycline on testicular tissue and sperm parameters in CD1 outbred mice [7]. According to them, the treatment of male mice with doxycycline in puberty led to long-lasting effects on reproductive organs and spermatozoa in adult males. They reported that the effect of doxycycline was concentration dependent. In addition, antitumor activities of doxycycline against different types of malignancies have also been reported elsewhere. For example, Sun et al. and Liu et al. have reported cytotoxicity and antimetastatic activity of doxycycline in melanoma and breast carcinomas [8,9]. According to Son et al., doxycycline has potential to show apoptotic activities in pancreatic cancer cells [10]. Duivenvoorden et al. has reported that doxycycline treatment could be effective to reduce the tumor burden in bone metastasis mouse model of human breast cancer [11]. All these studies suggest that this valuable antibiotic also has potential to treat other types of human cancers and thus a candidate anticancer drug of high research value. It is, therefore, important to monitor the concentration of doxycycline (doxy) in blood to optimize the dosage and reducing the side effects.

Currently, methods used for doxycycline detection involve analytical techniques such as high-performance liquid chromatography (HPLC) [12], sequential injection chromatography (SIC) [13], and potentiometry [14]. These techniques provide accuracy and reasonably good detection limits but have disadvantages such as need of complicated sample preparation, trained personnel, and sophisticated instruments and thus cannot provide onsite and fast detection. Therefore, there is a need to develop alternative methods that can provide onsite, fast, and sensitive detection of doxycycline.

Surface plasmon resonance (SPR) biosensor is an optical technique that measures the binding events quantitatively in real time without labeling the interacting molecules [15]. The physical principle of SPR technique involves the measurement of changes in refractive index when the interaction of molecules takes place at the sensor surface [16]. The benefits of the SPR technique include label-free detection, high sensitivity, real-time monitoring, and crude sample analysis [17]. These advantages make this SPR technique a reliable and convenient one to examine the binding specificity and interaction of biomolecules, as first reported in 1982 when Liedberg et al. initially reported the use of a SPR-based biosensor for the detection of biomolecular interaction [18]. Till now, this technique has been primarily used as an effective tool for biomolecular interaction analysis, but more recently, clinical analysis is increasingly reported [19]. SPR sensing proceeds without altering or damaging the composition of original analyte [20] and is increasingly proposed for clinical diagnostics [21,22], drug monitoring [23], environmental monitoring [24,25], food analysis [26,27], and biochemistry [28].

Despite the many advantages of SPR biosensors, the binding of small molecules to the sensor surface typically results in small shifts, which constitutes a limitation of SPR biosensors. Most portable and small SPR instruments are not sensitive enough to assess such small refractive index changes, which make them unfit to use for ultrasensitive detection of small organic drugs [29]. To overcome this limitation, different groups have employed various enhancement strategies in conjugation with SPR, such as enzyme [30], polymerase chain reaction (PCR) [31], and gold nanoparticles (AuNPs) enhancement methods [32]. Among them AuNPs-based enhancement strategies have received considerable attention and played a significant role in response amplification of SPR biosensors [33]. The ease of synthesis, good stability, biocompatibility, low toxicity, and ability of surface functionalization of AuNPs make them an attractive tool for biomedical applications [34]. AuNPs support localized surface plasmon resonances (LSPR), arising from the combined oscillations of electrons present in the conduction band of the metal [35]. The electronic coupling between the LSPR of gold nanostructures and SPR is often applied as a strategy to amplify the response signals of biosensors. For example, this strategy has been designed for the detection of methotrexate [23] and testosterone [36].

AuNPs are extensively applied in SPR biosensors, as such the effect of size of nanoparticles on SPR interactions is increasingly well understood. According to Kelly et al., size and shape of the nanomaterials could be effectively used to control the plasmonic characteristics as well as the electromagnetic field amplification of AuNPs [37]. Comparatively, larger nanostructures give higher sensitivity towards changes in refractive index than the smaller nanostructures. Uludag and Tothill studied the effect of size of AuNPs over the SPR sensor response. According to them, increasing the size of AuNPs resulted in higher sensor response [38]. Springer et al. observed that the size of AuNPs affects the diffusion mass transfer rate as well as the SPR signal and resulted in optical enhancement of SPR biosensor [39]. All these considerations suggested that the size of AuNP is critical and needs more attention while studying the biomolecular interaction via SPR biosensor.

To further build on the use of AuNPs-enhanced SPR for drug detection, we report on the fabrication of a doxy-AuNPs-based SPR biosensor for fast and sensitive detection of doxycycline. The use of the analyte to trigger AuNPs overgrowth is used as a novel sensing principle, where the signal of the SPR sensor is proportional to the concentration of doxycycline, in opposition to the usual competition assays resulting in a reduced response of the SPR sensor with concentration. Synthesized doxy-AuNPs were characterized by UV–VIS, X-ray diffraction (XRD), FT-IR, and transmission electron microscopy (TEM). SPR analysis was performed to demonstrate the detection of doxycycline. Various conditions were optimized to improve the SPR response. In this study, doxy-AuNP containing sodium chloride (NaCl) was employed as reagent providing further increase in the biosensor response. Thus, the doxy-AuNPs-based SPR biosensor is an excellent platform for the ultrasensitive detection of doxycycline.

## 2. Experimental

### 2.1. Materials

Gold (III) chloride trihydrate, doxycycline hyclate, N-hydroxysuccinimide (NHS), 16-mercapto-hexadecanoic acid (16-MHA), 11-mercapto-1-undecanol, ethanolamine hydrochloride, and sodium chloride were purchased from Sigma Aldrich (Oakville, Canada). Sodium hydroxide and N-ethyl-N’-(3-dimethylaminopropyl)-carbodiimide (EDC) were purchased from Fluka chemicals. Protease-activated receptor (PAR-1) was purchased from Cedarlane (Burlington, Canada).

### 2.2. Preparation of the Doxy-AuNPs

**Figure 1:**
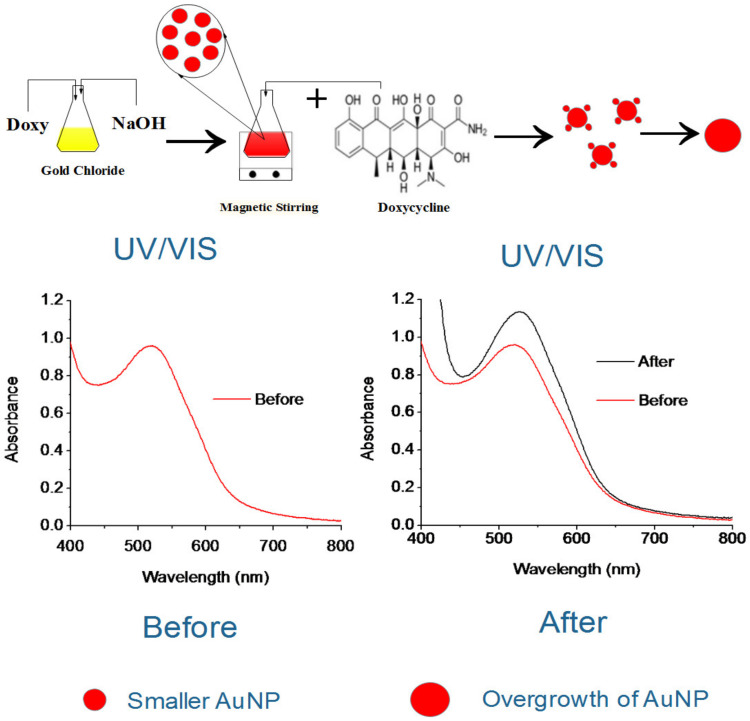
Scheme illustration of doxycycline effect on overgrowth of doxycycline Au nanoparticles (doxy-AuNPs).

To prepare doxy-AuNPs, aqueous solutions of 4 mL gold chloride (0.4 mM) and 2 mL doxycycline (0.8 mM) were added in a conical flask. To the mixture, 3 mL sodium hydroxide (0.01 M) was added. The mixture was continuously stirred for 3 min. After 3 min, a ruby red color was observed (Figure 1). A UV–Vis spectrophotometer (Model Cary 100 Bio, Varian, Palo Alto, USA) was used to monitor the synthesis of doxy-AuNPs in wavelength range of 300–800 nm.

### 2.3. Characterization of the Doxy-AuNPs

The crystalline nature of doxy-AuNPs was confirmed via XRD studies. The solution containing doxy-AuNPs was centrifuged three times at 10,000 rpm for 30 min and washed with deionized water each time. Then, the pellet obtained after centrifugation was left overnight to dry under fume hood. Powder XRD analysis was performed with Bruker D2 Phaser using Cu Kα radiation (1.54 Å λ) in the 2θ region, from 0 to 80°. Transmission electron microscopy (TEM) (FEI Tecnai t12) with voltage 80 kV and final emission 10 µA was used to examine the size and shape of doxy-AuNPs. Moreover, 2k AMT camera was applied to take micrographs of doxy-AuNPs. Sample for TEM was prepared by placing 10 µL of doxy-AuNPs colloidal solution on copper grid coated with carbon and formvar film. The sample was left for 24 h to dry and then analyzed with TEM. The FT-IR is the most widely used technique to study the interaction of biomolecules with the nanomaterials. The appearance of certain spectral changes enables to identify the interaction of certain functional groups with the nanomaterials [40]. The doxy-AuNPs were also characterized with FT-IR. The solution containing doxy-AuNPs was centrifuged three times at 10,000 rpm for 30 min and washed with deionized water each time. Then the pellet obtained after centrifugation was dried under fume hood for 24 h, transferred, and analyzed with FT-IR.

### 2.4. Fabrication of the SPR Sensor

SPR measurements were performed on a portable SPR instrument (Affinité Instruments, Montréal, QC, Canada) [23]. A dove prism with a gold film (1 nm Cr and 45 nm Au) was immobilized with a self-assembled monolayer (SAM) of 16-mercaptohexadecanoic acid and 11-mercapto-1-undecanol (Supporting Information, page S2). The SAM has the ability to bind receptor (protease-activated receptor-1) PAR-1 and is capable for resisting nonspecific adsorption on sensing surface for quantification of biomolecule [41]. This modified gold-coated prism with SAM was placed into the chip holder of the SPR setup. Then, a disposable PDMS flow cell was mounted over the prism and tighten with a clamp. The reference and sample solutions were injected at different sensing areas via separate injection ports. In PDMS flow cell, there are two separate flow channels, one for sample solution and other for reference solution. For the sample solution, the flow channel is S-shaped and comprise three different sensing areas, providing analysis of sample in triplicate. The total volume of the channel for sample analysis is 16 μL. For the reference solution, the flow channel covers the fourth sensing area with a volume of 5 μL. The whole SPR system was connected to custom LabView software via a laptop. Data acquired by the SPR system was controlled by software and minimum finding algorithm based on a second-order polynomial fit was used to integrate SPR signal at each time point. The sensorgrams for all four sensing areas were recorded in real time.

### 2.5. Immobilization of Receptor on Sensor Surface

In all SPR experiments, the SAM-modified gold-coated prism was inserted in the SPR instrument. First, Milli-Q water was added into the flow cell and left for 15–20 min for stabilization. Afterwards, the sensing surface was activated with EDC/NHS and left for 5 min until the resonant wavelength was constant. Then, the sensing surface was rinsed with PBS, followed by the injection of the receptor solution of protease-activated receptor-1 (PAR-1) at 5 μg mL^−1^ and reacted for 15 min. The receptor PAR-1 was covalently attached to the SAM through activated carboxylic acid group from EDC/NHS. Subsequently, nonspecific binding sites on the sensing surfaces were blocked by injecting 1 M ethanolamine hydrochloride (pH 8.0) for 10 min followed by rinsing with PBS to remove noncovalently attached receptor PAR-1. This procedure was repeated for all SPR experiments.

### 2.6. Electrolytic Stability of Doxy-AuNPs

To examine the electrolytic stability of doxy-AuNPs, different concentrations of NaCl (from 50 to 1000 mM) were added in doxy-AuNPs colloidal solutions and their UV spectra were recorded (Model Cary 100 Bio, Varian, Palo Alto, USA) in the wavelength range of 300–800 nm. To select the suitable electrolytic condition of doxy-AuNPs, which can give larger SPR response, doxy-AuNPs containing varying concentrations of NaCl (from 50 to 1000 mM) were injected in SPR followed by rinsing each time before and after each injection with same concentration of NaCl in water to record the refractive index baseline. A control experiment of doxy-AuNPs without NaCl was also conducted and sensorgrams were recorded in real time.

### 2.7. Sequential Analysis for Determination of Concentration of Doxycycline

To examine the effect of doxycycline on the growth of synthesized doxy-AuNPs, varying concentrations of doxycycline (from 1 nM to 1 mM) were added in suspensions of doxy-AuNPs and their UV spectra were recorded in the wavelength range of 300–800 nm. Furthermore, to carry out detection of doxycycline with the SPR system, varying concentrations of doxycycline (0.1 nM to 100 μM) were added in the colloidal suspension of doxy-AuNPs and injected sequentially in flow cell at room temperature for 30 min followed by rinsing each time before and after each injection with 100 mM NaCl in water for 5 min to record baseline. Interaction between biological receptor PAR-1 and doxy-AuNPs was measured as binding shift by SPR biosensor in real time. Control experiments were performed by injecting doxy-AuNPs (containing optimized NaCl concentration) without adding free doxycycline in SPR system. Origin software was used to process the data utilizing the minimum wavelength finding algorithm. From the sensorgram, last 50 data points of 100 mM NaCl steps before and after the doxycycline sensing steps were used to calculate the binding shift from sensorgram. The logarithm of doxy concentration was plotted against the binding shift to find the correlation between concentration of doxycycline and binding shift. Triplicate measurements were carried for all conditions. Reproducibility was obtained from the triplicate SPR measurements of 100 nM doxycycline and measured as a coefficient of variation resulting from the ratio of the standard deviation and the mean response, in percentage.

## 3. Results and Discussion

### 3.1. Strategy of the Assay

Doxycycline is a broad-spectrum drug with its antimicrobial as well as antitumor activities [4,8,10,11]. PAR-1 is the receptor protein with which doxycycline binds to inhibit the tumor progression [6]. Therefore, SPR analysis for doxycycline detection mainly depends upon the interaction of doxy-AuNPs with PAR-1 immobilized on sensor surface. The interaction of doxy-AuNPs with PAR-1 resulted in change of refractive index, which consequently gives SPR response in the form of wavelength shift. In this work, doxy-AuNPs containing NaCl were employed as an amplification element to enhance the SPR response. Furthermore, addition of free doxycycline in doxy-AuNPs colloidal solution causes overgrowth of doxy-AuNPs, which consequently further enhances the SPR biosensor response. The concentration of doxy was correlated with biosensor response. This concept could be applied to other small molecules that can act as reducing agents for gold ions. It is advantageous of synthesizing the AuNP with the analyte as it provides a simple method for synthesis and functionalization in a single step and allowed for the efficient overgrowth of the AuNP in presence of the analyte.

### 3.2. Synthesis and Stability of Doxy-AuNPs

**Figure 2:**
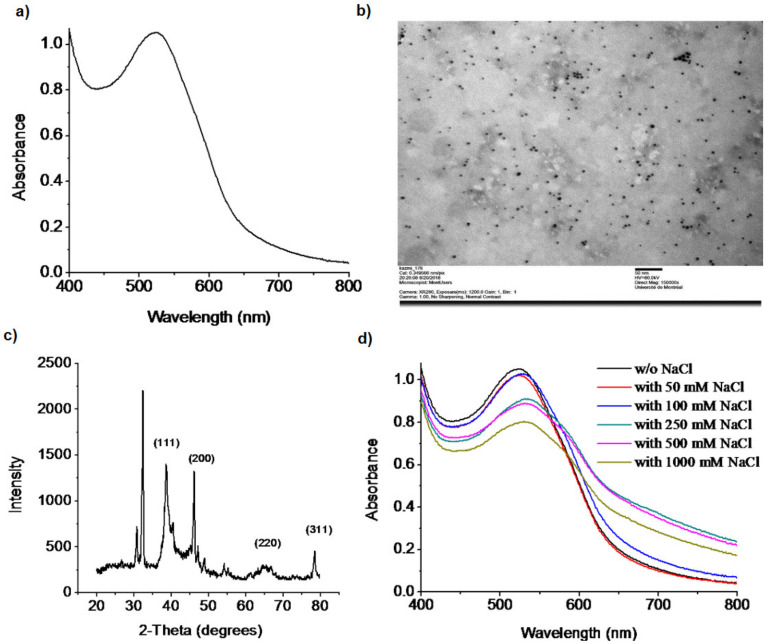
(**a**) UV–Vis spectrum of doxy-AuNPs. (**b**) TEM image of doxy-AuNPs, acquired at 80 kV, with exposure of 1200 ms and magnification of 150,000X. The scale bar represents 50 nm. (**c**) XRD spectra of doxy-AuNPs. (**d**) UV–Vis spectra showing the electrolytic stability of doxy-AuNPs in the presence of different concentrations of NaCl.

A wet chemical reduction method was used to synthesize the doxy-AuNPs [42]. Doxycycline, being a polyphenolic compound, reduces and stabilizes the AuNPs. The polyphenolic groups are oxidized to their respective quinones by AuCl_4_^−^ with H^+^ transfer and AuNPs formation. Recently, He et al. has reported the similar phenomena [43]. The b-agonists that possess monophenolic group or aniline showed the capability of reducing HAuCl_4_ into AuNPs at elevated temperature, which means the reducing capability of such compounds are weaker than the one of the polyphenolic compounds, such as doxycycline. Immediately after addition of doxycycline and sodium hydroxide in gold chloride solution, AuNPs were synthesized in 3 min with characteristic ruby red color, similar to another synthesis previously reported for L-methionine [44]. Synthesis of doxy-AuNPs was confirmed using UV–Vis spectrophotometry. A sharp surface plasmon resonance was observed at 520 nm, which is a characteristic LSPR (localized surface plasmon resonance) in spherical gold nanoparticles [45], thus confirming the synthesis of doxy-AuNPs (Figure 2a). TEM images were then acquired for further supporting the synthesis of doxy-AuNPs. Homogenously distributed spherical gold nanoparticles were obtained with this method (Figure 2b). Average particle size of doxy-AuNPs calculated was 4.7 ± 0.7 nm (n = 169, histogram in Supporting Information, Figure S1). The crystalline nature of doxy-AuNPs was examined through XRD analysis. XRD pattern of doxy-AuNPs showed strong diffraction peaks at 38.5°, 46.1°, 64.5°, and 78.6° corresponding to {111}, {200}, {220}, and {311}, respectively, which reflects the crystalline nature of doxy-AuNPs (Figure 2c) [46].

To examine the electrolytic stability of synthesized doxy-AuNPs, different concentrations of NaCl (from 50 to 1000 mM) were added in doxy-AuNPs colloidal solutions and their UV spectra were recorded. Doxy-AuNPs showed good electrolytic stability with slight red shift (from 520 to 530 nm) and small decrease in intensity up to 100 mM NaCl, whereas in case of higher concentrations from 250 to 1000 mM NaCl, LSPR band of doxy-AuNPs further red shifted from 530 to 540 nm with significant broadening and decrease in intensity (Figure 2d). It was likely due to the refractive index shift of high salt solution and screening of surface charge on the AuNP. Srivastava and Gupta have also reported this phenomenon. According to them, resonance wavelength increases with higher salt content due to interaction of the salts with the metal film [47]. The effect of salt has been previously shown to have an impact on the Debye length and the interaction of ligand-modified AuNP for a methotrexate SPR sensor [48]. As such, the stability of the doxy-AuNP was essential to carry the SPR measurements reported in this paper.

**Figure 3:**
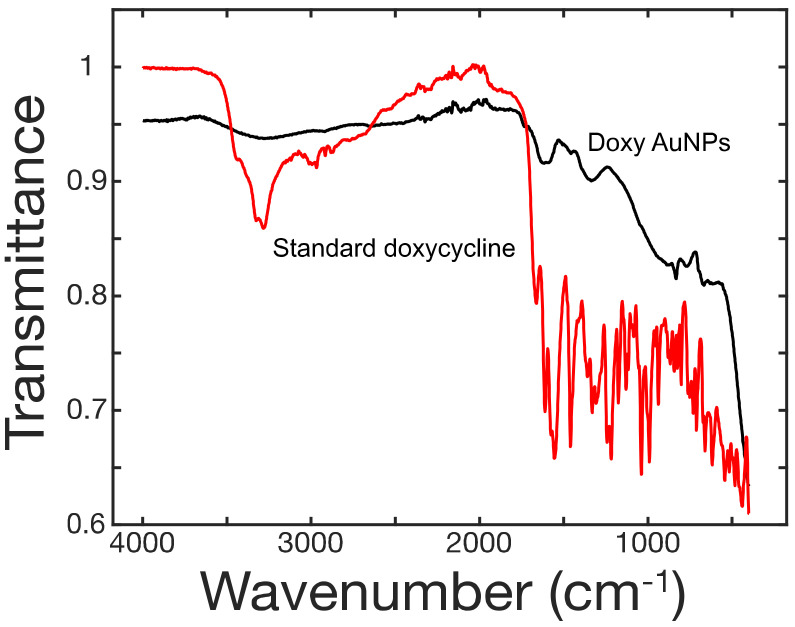
FT-IR spectra of doxycycline (red) and doxycycline-modified gold nanoparticles (black).

The interaction of doxycycline in synthesis of AuNPs was studied through FT-IR (Figure 3). In case of pure doxycycline, the absorption band was observed in the range of 3400–3200 cm^−1^ for O-H and N-H bonds but this band completely disappeared in case of doxy-AuNPs. This observation hints about the involvement of these moieties in the modification process. Likewise, the absorption bands in the range of 1700–1500 cm^−1^ were attributed to α,β-unsaturated carbonyl of amide and ketone functionalities of doxycycline. These signals moved to higher values in case of doxy-AuNPs, although the intensity of signals is low. The shifting to higher values justified the involvement of hydroxyl group resulting into disappearance of C=C conjugation.

### 3.3. SPR Analysis for Detection of Doxycycline

#### 3.3.1. Optimization of Electrolytic Conditions of Doxy-AuNPs

**Figure 4:**
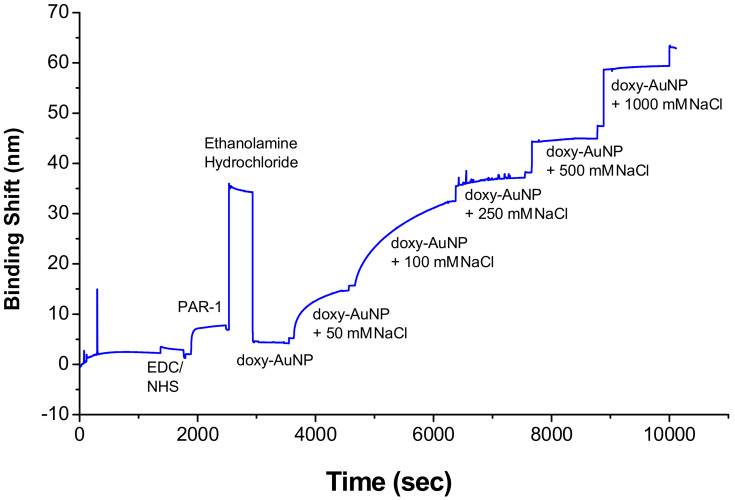
Effect of sodium chloride (NaCl) concentration on the surface plasmon resonance (SPR) response of doxy-AuNPs. The SPR response was measured in the Kretschmann configuration, and the binding shift refers to the propagating plasmon of the gold film.

A shorter Debye length and, as a consequence, decreased colloidal stability are required for the molecular interaction of target analyte to occur on a surface-bound receptor. The presence of NaCl causes the electrostatic screening of surface charges by dissolved ions and reduces the Debye length, and resulted in the higher SPR response reported previously for a methotrexate assay [48]. To select the suitable electrolytic condition of doxy-AuNPs, which can give higher SPR response, doxy-AuNPs without NaCl and with varied concentrations of NaCl (from 50 to 1000 mM) were injected in SPR system. In the absence of NaCl, very low and undetectable SPR response was observed for doxy-AuNPs, whereas significantly higher SPR response was observed for doxy-AuNPs in the presence of NaCl (Figure 4 and Figures S2 and S3 in Supporting Information page S3). Very low SPR response of doxy-AuNPs is mainly attributed to the small size (4.7 nm) and larger Debye length of doxy-AuNPs in absence of salt, which makes them unfit to enter deep in the binding pocket of receptors bound to the sensor surface [49,50]. On the contrary, addition of NaCl to doxy-AuNPs causes the electrostatic screening of surface charges by dissolved ions and reduces the Debye length, and thus resulted in the largest SPR response [48]. The control with the same NaCl concentration before injection of the doxy-AuNP led to changes in SPR shifts much smaller (i.e., 1 nm) than with the doxy-AuNP. Doxy-AuNP with 50 mM NaCl led to a SPR shift of 9.5 nm, whereas the doxy-AuNP with 100 mM NaCl provided a 25 nm SPR shift. Since the doxy-AuNPs containing 100 mM NaCl generated maximum SPR response signal (Figure 4), these conditions were selected for all further SPR bioassays for detection of doxycycline.

#### 3.3.2. SPR Bioassay for Doxycycline Detection

**Figure 5:**
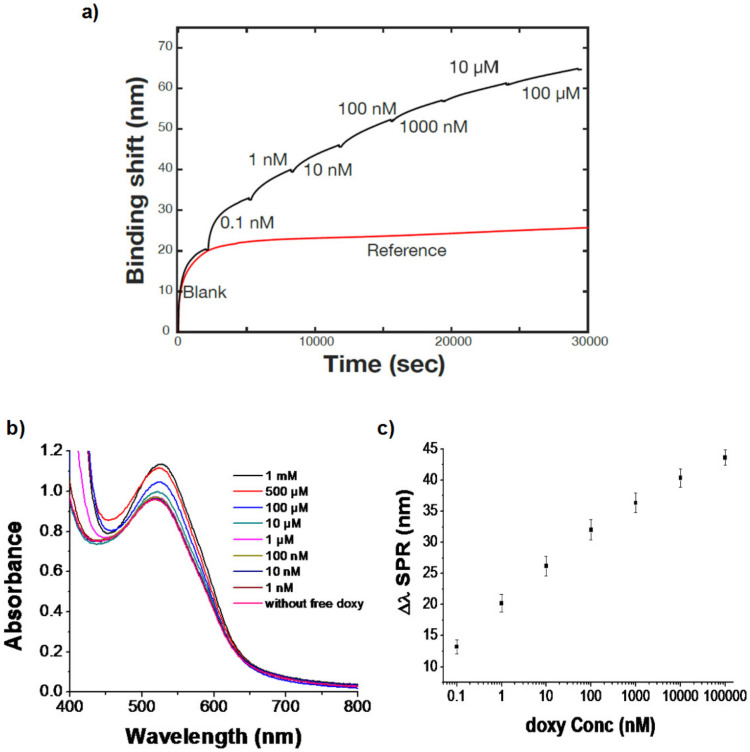
(**a**) Propagating SPR response of doxy-AuNPs (control, red trace) and of doxy-AuNPs with varying concentrations of doxy (analyte, black trace). (**b**) UV–Vis spectra indicating the effect of addition of doxycycline on growth of doxy-AuNPs. (**c**) Sequential binding curve presenting a correlation between log of doxy concentration and SPR response. Error bars indicate standard deviation of triplicate measurements.

Sequential SPR analysis was performed for the detection of doxycycline (analyte). As the Au salt and doxycycline were reacted in equimolar conditions, it is hypothesized that the reaction is incomplete and thus, leaves unreacted Au ions in solution in addition to the AuNPs. Different concentrations of doxycycline (0.1 nM to 10 μM) were added in the colloidal solution of doxy-AuNPs (containing 100 mM NaCl) and injected sequentially into the flow cell of SPR system. In comparison, a control sample was run in which doxy-AuNPs (containing 100 mM NaCl) were injected in flow cell of SPR system. For the control sample, a 25 nm binding shift was observed with doxy-AuNPs (Figure 5a, red trace) without the addition of doxycycline in solution while an increase in binding shift was observed with successive addition of increasing concentrations of free doxycycline in the doxy-AuNPs colloidal solutions (Figure 5a, black trace). This enhancement of SPR response on successive addition of free doxycycline is mainly because increased concentration of doxycycline resulted in rapid growth of doxy-AuNPs, similar to reported elsewhere for other tetracyclines [50], which consequently give higher SPR response.

We conclude that the growth was likely due to the already present doxy-AuNPs in colloidal solution working as nuclei for the gold atoms remaining from the AuNP synthesis and doxycycline (analyte). This caused the rapid growth of seeds of doxy-AuNPs (Figure 1), which consequently resulted in higher SPR response. Our UV–Vis results showing the effect of doxycycline addition in doxy-AuNPs colloidal solution also supports this overgrowth. On addition of varying concentrations of doxycycline (from 1 nM to 1 mM) in doxy-AuNPs colloidal solution, the LSPR of doxy-AuNPs showed slight red shift with increase in intensity (Figure 5b), a typical feature of nanoparticle growth. Shen et al. has also reported the similar phenomena for effect of tetracycline addition to the *in situ* growth mechanism of AuNPs. According to them, AuNPs seeds (citrate stabilized) present in the solution work as nuclei for the conjugation and growth of gold atoms produced as result of reaction between tetracycline and HAuCl_4_ [50]. Furthermore, a similar effect of size of AuNPs on SPR response was reported by Bukar et al. According to them, gold nanoparticles with large size and smaller Debye length on the SPR sensor surface prevail over interaction with surface-bound receptors and lead to a higher SPR response [48].

Calibration of this SPR sensor shows linearity over several orders of magnitude and detection of doxycycline from nanomolar to micromolar (Figure 5c), which is in the clinical range, demonstrating the applicability of the sensor for doxycycline. The blood serum levels of doxycycline reach 1–10 mg/mL within about 2 h after administration of a dose, which corresponds to approximately 2–22 µM [51]. The sensor also showed high reproducibility with a coefficient of variation around 5% and a limit of detection of 7 pM (established from the sensitivity and noise level of the sensor). This low limit of detection compared favorably to the other analytical techniques developed earlier for doxycycline detection (Table 1) and therefore highlights the advantages of the SPR sensor for doxycycline. In addition to the excellent analytical performance, the SPR sensor is relatively simple to use on a portable instrument that would be conducive for point-of-care measurements.

## 4. Conclusions

An ultrasensitive SPR biosensor based on doxy-AuNPs has been successfully developed for the detection of doxycycline. Variation in size and growth of doxy-AuNPs affected the biosensor response. To overcome the limitation of low molecular weight of analyte, doxy-AuNPs (containing 100 mM NaCl) have been applied as an amplification element to obtain significantly enhanced SPR response. The reported biosensor allowed for the detection of doxycycline as low as 7 pM. The high sensitivity, low limit of detection, excellent signal response time (less than half an hour), good stability, and reproducibility make this SPR biosensor an excellent alternative to the conventional methods for the detection of doxycycline. In future, the present biosensor can be applied in different fields of medical diagnostics and environmental monitoring for the detection of doxycycline.

## Figures and Tables

**Table 1:** Comparison with other analytical techniques developed for doxycycline detection.

Ref.	Method	Linearity Range	Limit of Detection (LOD)	
			As Reported in Paper	Value in mol/L
Jeyabaskaran et al., 2014 [52]	RP-HPLC	25–150 µg/mL	0.02 µg/mL	3.7 × 10^−8^ mol/L
Adrian et al., 2012 [53]	ELISA	0.25–6.7 µg/mL	0.1 µg/L	1.95 × 10^−10^ mol/L
Kogawa et al., 2012 [54]	HPLC–UV	50–100 µg/mL	2.83 µg/mL	5.2 × 10^−6^ mol/L
Selvadurai et al., 2010 [55]	Liquid chromatography–mass spectrometric (LC-MS)	0.5–5 µg/mL	50 ng/mL	9.2 × 10^−8^ mol/L
Ramesh et al., 2010 [56]	RP-HPLC	30–300 µg/mL	0.02 µg/mL	3.7 × 10^−8^ mol/L
This work	Surface plasmon resonance biosensor	0.1 nM–100 µM	7 pM	7 × 10^−^^12^ mol/L

## Supplementary Materials

The following are available online at https://www.mdpi.com/2079-6374/10/11/184/s1, Figure S1: Histogram of doxy-AuNPs, Figure S2: SPR sensorgram indicating the response of doxy-AuNP (without NaCl) towards the doxycycline determination, Figure S3: SPR sensorgram indicating the response of doxy-AuNP (with and without NaCl) towards the doxycycline determination and additional information on the fabrication of SPR sensor using 16-mercaptohexadecanoic acid (16-MHA) and 11-mercapto-1-undecanol, on Protease activated receptor 1 (PAR-1) and on the role of NaCl in SPR sensing of doxycycline
