# Supplementary material for: Drug-Based Gold Nanoparticles Overgrowth for Enhanced SPR Biosensing of Doxycycline
Source: Biosensors (Basel). 2020 Nov 19;10(11):184. doi: 10.3390/bios10110184 (PMC7699512; doi:10.3390/bios10110184)
Supplement: Supplementary file 1 [file biosensors-10-00184-s001.pdf]

biosensors

MDPI

Article

Drug-Based Gold Nanoparticles Overgrowth for Enhanced SPR Biosensing of Doxycycline

Syed Akif Raza Kazmi [1,2,*], Muhammad Zahid Qureshi [1] and Jean-Francois Masson [2,*]

[1] Department of Chemistry, Government College University Lahore, Lahore 54000, Pakistan; dr.zahidqureshi@gcu.edu.pk

[2] Département de chimie, Québec Centre for Advanced Materials and Regroupement Québécois sur les Matériaux de Pointe, C.P 6128 Succursale Centre-Ville Université de Montréal, Montreal, QC H3C 3J7, Canada

* Correspondence: akifr1@gmail.com (S.A.R.K.); Jf.masson@umontreal.ca (J.-F.M.)

Received: 22 October 2020; Accepted: 18 November 2020; Published: 20 November 2020

Supporting information content:

Figure S1. Histogram of the AuNP size (p. S-2)

Figure S2. Sensorgram of the AuNP interaction in absence of salt (p. S-4)

Figure S3. Sensorgram of the AuNP in presence of salt (p. S-5)

Further experimental details on chip formation (pp. S-2 to S-3)

Histogram of doxy-AuNPs

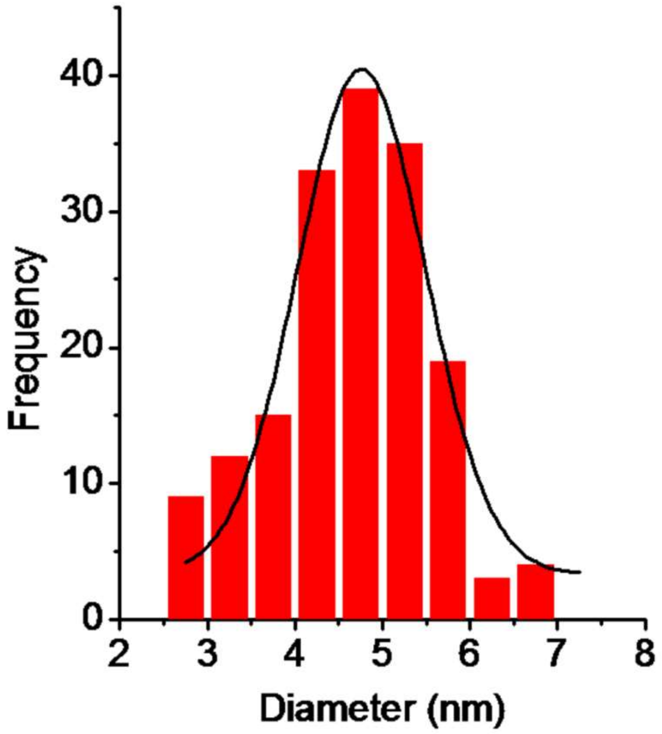

Figure S1. Histogram of doxy-AuNPs

Fabrication of SPR sensor using 16-mercaptohexadecanoic acid (16-MHA) and 11-Mercapto-1-undecanol

The dove prism was coated with 1 nm Cr and 45 nm Au (ESPI metals) utilizing a Cressington 308R sputter coater. Afterwards, the gold coated dove prism was dipped into solution of 0.1 mM 16-mercaptohexadecanoic acid and 0.9 mM 11-mercapto-1-undecanol and left overnight for the formation of self assembled monolayer (SAM) of 16-mercaptohexadecanoic acid and 11-mercapto-1-undecanol. After that, the SAM modified gold coated prism was thoroughly rinsed 3 times with ethanol and purified water and dried under nitrogen.

Afterwards, the sensing surface was activated with 20 mM *N*-ethyl-*N*′-(3-dimethylaminopropyl)-carbodiimide (EDC, Fluka, purity > 97%) and 5 mM *N*-hydroxysuccinimide (NHS, Sigma Aldrich, purity > 98%) and left for 5 min. Then, the sensing

surface was rinsed with PBS, followed by the injection of the receptor solution of Protease Activated Receptor-1 (PAR-1) at 5 µg mL^{-1} and reacted for 15 min.

The receptor PAR1 was covalently attached with the SAM through activated carboxylic acid group from EDC/NHS. Subsequently, non-specific binding sites on sensing surface were blocked by injecting 1 M ethanolamine hydrochloride (Sigma Aldrich, ≥ 99.0%) for 10 min adjusted to pH 8.0 with 10 M sodium hydroxide (Fluka, purity > 98%) followed by rinsing with PBS to remove non-covalently attached receptor PAR1. This procedure was repeated for all SPR experiments.

Protease Activated Receptor (PAR-1)

The protease activated receptor (PAR-1) protein was received from Cedarlane and stored at -80°C until use. One the day of experiment, PAR-1 was dissolved in acetate buffer (20 µg/ 80 µL, m/v) adjusted at pH 8. Then, 10 µL of the PAR1 solution was taken from this stock PAR1 solution. To this, 490 µL acetate buffer (adjusted at pH 8) was added. This dilution of PAR-1 with final concentration 5 µg/mL was used for each SPR biosensor experiment.

Role of NaCl in SPR sensing of doxycycline

For SPR sensing of doxycycline, the role of sodium chloride was very important to maximize wavelength shift. It was interesting to note that doxy-AuNPs in absence of salt provided a very low wavelength shift (almost negligible) (Figure S2). We hypothesize this is due to a combination of the very small NP size (*i.e.* 4.7 nm diameter) and screening of charges in the low salt concentrations. Furthermore, a control test with NaCl solution in water (before injection of doxy-AuNPs) also led to a very small wavelength shift of 1 nm (Figure S3). However, doxy-AuNPs in the presence of NaCl provide a significantly larger wavelength shift (Figure S3). With 50 mM NaCl, doxy-AuNPs give the wavelength shift of 9.5 nm and with 100 mM NaCl, doxy-AuNPs give 25 nm wavelength shift (Figure S3). During the optimization for NaCl concentration, the highest wavelength shift was obtained at 100 mM

NaCl. This was the reason that doxy-AuNPs with 100 mM NaCl were used for all further SPR experiments for doxycycline determination.

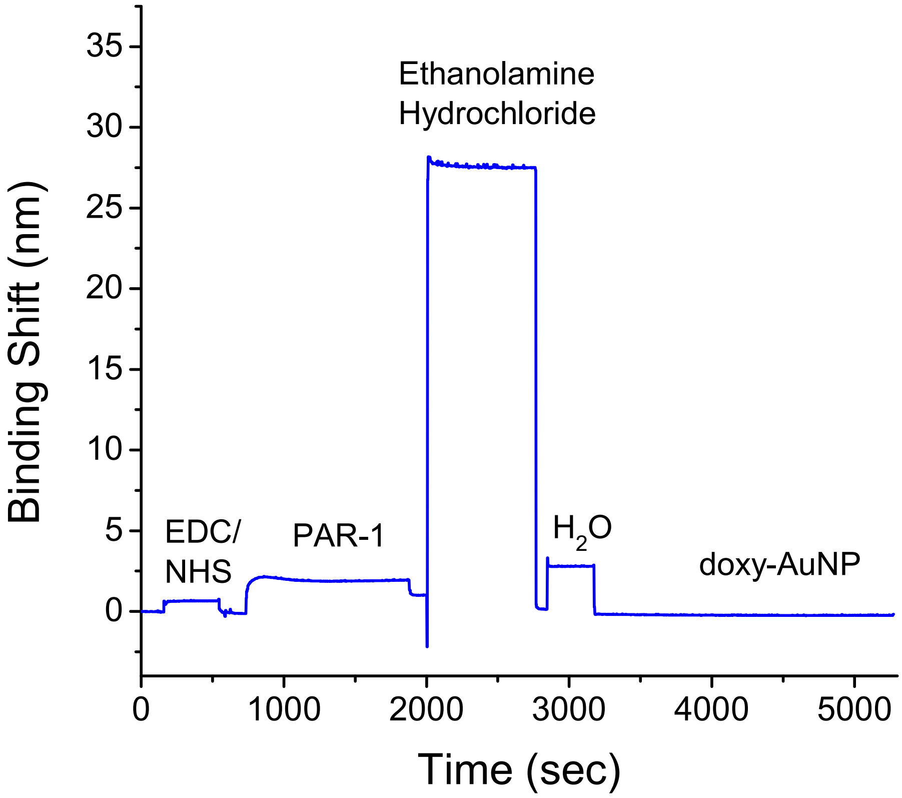

Figure S2. SPR sensorgram indicating the response of doxy-AuNP (without NaCl) towards the doxycycline determination

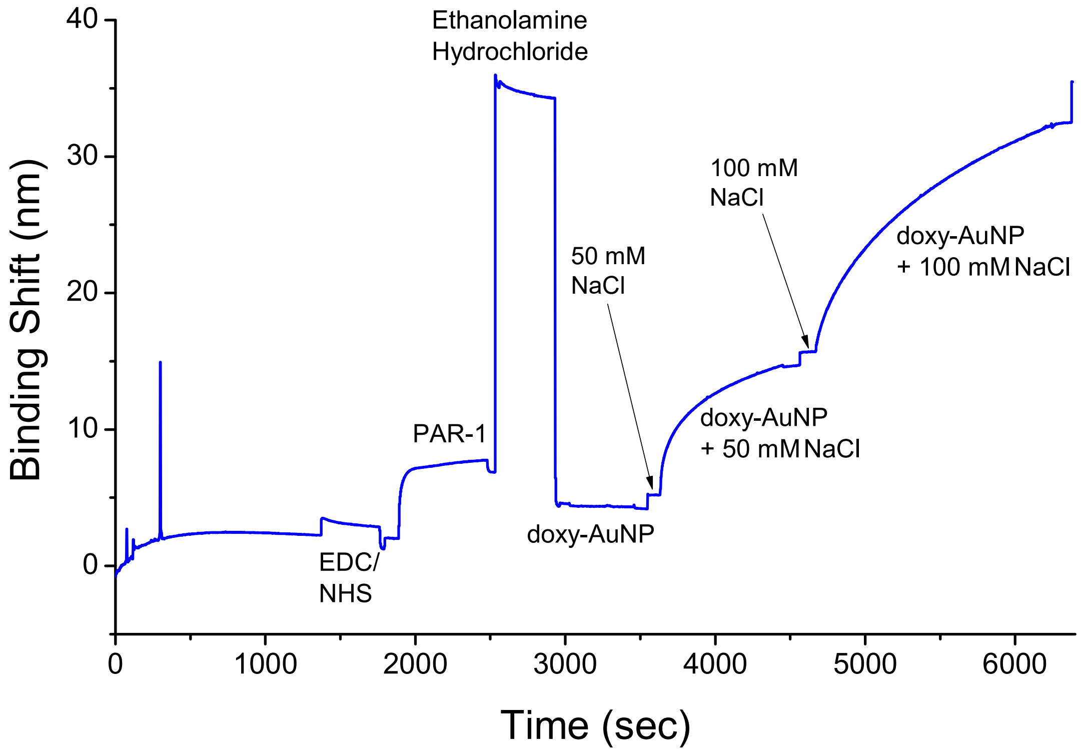

Figure S3. SPR sensorgram indicating the response of doxy-AuNP (with and without NaCl) towards the doxycycline determination
